# Age-dependent thrombin generation predicts 30-day mortality and symptomatic thromboembolism after multiple trauma

**DOI:** 10.1038/s41598-023-28474-7

**Published:** 2023-01-30

**Authors:** Maj Lesbo, Claus V. B. Hviid, Ole Brink, Svend Juul, Lars C. Borris, Anne-Mette Hvas

**Affiliations:** 1 Department of Ortopedic Surgery, Aarhus University Hospital, 8200 Aarhus N, Denmark; 2 Department of Clinical Medicine, Aarhus University, 8000 Aarhus C, Denmark; 3 Department of Clinical Biochemistry, Aarhus University Hospital, 8200 Aarhus N, Denmark; 4 Department of Clinical Biochemistry, Aalborg University Hospital, 9000 Aalborg, Denmark; 5 Department of Public Health, Research Unit for Epidemiology, Aarhus University, 8000 Aarhus C, Denmark

**Keywords:** Prognostic markers, Trauma, Ageing

## Abstract

Trauma-induced coagulopathy (TIC) is a risk factor for death and is associated with deviations in thrombin generation. TIC prevalence and thrombin levels increase with age. We assayed in vivo and ex vivo thrombin generation in injured patients (n = 418) to specifically investigate how age impacts thrombin generation in trauma and to address the prognostic ability of thrombin generation. Biomarkers of thrombin generation were elevated in young (< 40 years) and older (≥ 40 years) trauma patients. In vivo thrombin generation was associated with Injury Severity Score (ISS) and this association was stronger in young than older patients. In vivo thrombin generation decreased faster after trauma in the young than the older patients. Across age groups, in vivo thrombin generation separated patients dying/surviving within 30 days at a level comparable to the ISS score (AUC 0.80 vs. 0.82, *p* > 0.76). In vivo and ex vivo thrombin generation also predicted development of thromboembolic events within the first 30 days after the trauma (AUC 0.70–0.84). In conclusion, younger trauma patients mount a stronger and more dynamic in vivo thrombin response than older patients. Across age groups, in vivo thrombin generation has a strong ability to predict death and/or thromboembolic events 30 days after injury.

## Introduction

Trauma remains a leading cause of death world wide, and disturbances in hemostasis are closely associated with patient outcome^1^. In the acute phase of trauma, hemorrhage accounts for approximately 1/3 of the cases where the victims subcrumb to their injuries^2^, and 25–35% of severely injured patients demonstrate signs of coagulopathy on arrival to the trauma-resuscitation room^3–5^. At later stages after trauma, patients are at high risk of thromboembolic events^6^, and venous thromboembolism is a common cause of death and morbidity among patients having survived the initial 24 h^6–8^.

It has become increasingly clear that this trauma-induced coagulopathy (TIC) cannot be explained solely by the classic lethal triade, but rather is a complex physiological reponse to the injury that may be aggrevated by hemodilution, acidosis and hypothermia^4,9^. The coagulation response in TIC may be characterized by hypocoagulation or hypercoagulation^9^. Furthermore, the hemostatic response alters along time after injury and physicians are challenged with the difficult task of balancing the immediate risk of bleeding against the risk of thromboembolic events. Previous studies have demonstrated that on arrival at hospital, the majority of patients present biochemical evidence of hypercoagulation, whereas only a smaller fraction has a decreased thrombin generation^10,11^. Patients with severe hypocoagulation are more likely to require massive blood transfusions and, paradoxically, are at increased risk for late thromboembolic events^9–11^. Patient age is a well-established risk factor for TIC and/or post-trauma adverse events^12^. At the same time, evidence exsists to suggest that biological responses in elderly trauma patients are different from that of younger patients^13–15^.

Therefore, a need exists to further improve our understanding of the coagulation responses to trauma; how these changes are impacted by patient age and impact the risk for bleeding, thromboembolic events and death, and ultimately affect clinical decision-making. In this study, we investigate in vivo and ex vivo thrombin generation in a cohort of mixed trauma patients and address age-specific differences in the responses. Furthermore, we report on the association between 30-day mortality and the development of thromboembolic events.

## Results

### Study inclusion and selection process

**Figure 1:**
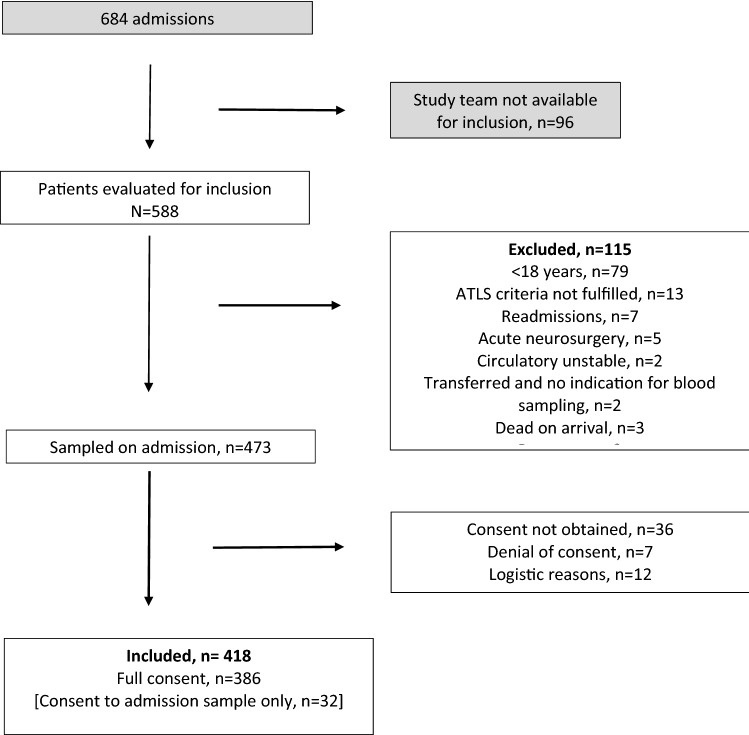
Study inclusion and selection process.

During the study period, 684 patients were admitted to our trauma center (Fig. 1). The study team was unavailable at the time of admission of 96 patients. These 96 patients were not different from the 588 that were evaluated for inclusion in respect to age, sex, or ISS on admission (*p* > 0.29, for all). Of the 588 patients evaluated for inclusion, 115 fullfilled at least one exclusion criterion and were excluded from participation in the study. Among these, children (< 18 years) comprised the vast majority, followed by patients not fulfilling the ATLS criteria. Seven were readmissions and two patients were transferred from lower level care centers late after the trauma and therefore not included. Only ten patients were excluded because the severity of their injuries precluded collection of study samples. Of these, five were directed for immediate neurosurgery, three patients were dead on arrival, and two patients were too circulatory unstable to allow the collection of study samples. The admission blood sample was obtained from the remaining 473 patients. Of these, 43 subsequently denied consent or consent could not be obtained for practical reasons. In 12 cases, logistic problems caused the exclusion of the patient. Thus, the process resulted in the inclusion of 418 trauma patients. Among these, 32 consented the use of the admission blood sample but refrained from further blood sampling. The remaining 386 patients provided full consent.

### Charateristics of the cohort

The characteristics of the included patients, the treatment course and outcomes are presented in Table 1. Males dominated the cohort. The use of platelet inhibitor and anticoagulant therapy was limited. Median time from injury to admission was below one hour, 12% percent of the patients were in circulatory shock on admission (shock index ≥ 0.8) and 25% were severely injured (ISS > 15).

**Table 1:** Study cohort characteristics, interventions within 72 h of hospital stay and 30-days outcome. All values are presented as n (%) unless stated otherwise.

	All patients	Younger (< 40 years)	Older (≥ 40 years)
Male/female	280/138 (67/33%)	111/64 (63/37%)	169/74 (70/30%)
Age, years (median, (iqr))	44 (34)	25 (11)	57 (22)***
Pre-admission medical treatment			
Platelet inhibitors	27 (6%)	1 (1%)	26 (11%)***
Anticoagulants	24 (6%)	0	24 (10%)
Vitamin-K antagonists	12 (3%)	0	12 (5%)
Direct oral anticoagulants	10 (2%)	0	10 (4%)
Unspecified	2 (0.5%)	0	2 (1%)
Antihypertensiva	57 (14%)	2 (1%)	55 (23%)***
Admission			
Primary admissions	385 (92%)	164 (94%)	221 (91%)
Intubated on admission	52 (12%)	18 (10%)	34 (14%)
Temperature < 35 °C, n = 99	5 (5%)	1 (1%)	4 (4%)
Shock Index (HR/systolic blood pressure) ≥ 0.8, n = 351	43 (12%)	20 (14%)	23 (11%)
Respiratory rate ≥ 25/min, n = 196	10 (5%)	3 (4%)	7 (6%)
Time from trauma to admission, median (iqr), min	55 (37–76)	50 (35–71)	60 (42–79)**
Trauma mechanism			
Blunt	364 (87%)	143 (82%)	221 (91%)**
Penetrating	17 (4%)	14 (8%)	3 (1%)**
Combined	4 (1%)	4 (4%)	0*
Unknown	33 (8%)	14 (8%)	19 (8%)
Trauma severity			
Injury severity score			
0–8	209 (50%)	112 (65%)	97 (40%)***
9–15	93 (22%)	27 (15%)	66 (27%)**
16–24	51 (12%)	13 (7%)	38 (16%)*
25–75	54 (13%)	16 (9%)	38 (16%)
Unknown	11 (3%)	7 (3%)	4 (2%)
Patients operated within 72 hours^1^			
One round of surgery	126 (30%)	44 (25%)	82 (34%)
Repeated rounds of surgery	28 (7%)	8 (5%)	20 (8%)
Surgical procedures within 72 hours^2^			
Orthopedic surgery	76 (32%)	27 (12%)	49 (21%)
Neuro surgery	43 (18%)	19 (8%)	24 (10%)
Spine surgery	27 (10%)	7 (3%)	20 (9%)
Thoracic surgery	26 (11%)	6 (3%)	20 (9%)*
General surgery	20 (9%)	7 (3%)	13 (6%)
Minor superficial procedures	19 (8%)	9 (4%)	10 (4%)
Interventional radiology	14 (6%)	3 (1%)	11 (5%)
Facial surgery	9 (4%)	6 (3%)	3 (1%)
Medical interventions within 72 hours^3^			
Blood transfusion	38 (9%)	11 (6%)	27 (11%)
Massive blood transfusions	15 (4%)	6 (3%)	9 (4%)
Thromboprophylaxis	146 (35%)	43 (25%)	103 (42%)***
Initiated before 72 h	123 (29%)	34 (19%)	89 (37%)***
Initiated after 72 h	23 (6%)	9 (5%)	14 (6%)*
30-days outcome			
Dead	15 (4%)	4 (2%)	11 (5%)
Any thromboembolic event	12 (3%)	0	12 (5%)**
Venous	7 (2%)	0	7 (3%)**
Arterial	5 (1%)	0	5 (2%)**

^1^A surgical intervention was defined as one or more surgical procedures performed on the same day. Repeated surgery was defined as two or more surgical procedures 24 h apart.

^2^Surgical procedures within 72 h is presented as the sum of procedures in each category and the percentage of the total number of procedures performed in the cohort. Minor superficial procedures include gastro- and bronchoscopy, fasciotomies, major wound revisions, otorhinolaryngology surgery other than skeletal, plastic surgery, eye surgery, urologic procedures, vascular surgery on lower extremity artery, and digital amputation. Interventional radiology group contain vascular coils, coronary angiography, thrombectomy and transcutaneous drains.

^3^Massive blood transfusion was defined as more than 10 units of red blood cells and/or fresh frozen plasma.

*/**/***: *p* < 0.05/< 0.01/< 0.001.

Within the initial 72 h after the injury, a total of 234 surgical procedures were performed on a total of 154 patients. In 28 cases repeated surgery was performed. Orthopedic procedures dominated and were followed by neuro surgery, spine surgery and thoracic procedures. Overall, 9% of the patients received in-hospital blood transfusions, of which 40% were massive transfusions. One third of the patients received thromboprophylaxis with low molecular weight heparin (LMWH) during their stay.

At 30 days, 31 patients were lost to follow-up. Among these, 29 patients had been transferred to hospitals not covered by our electronic medical chart, and two patients (0.5%) could not be accounted for. Among the remaining 387 patients, 15 patients had succumbed within a median of 69 h (24–159, iqr) after admission. Of these patients, 13 died during the stay and two after discharge to lower-level care. Seven venous and five arterial symptomatic thromboembolic events occurred within 30 days after admission. Four of the five patients with arterial events subsequently died, whereas all patients suffering venous thromboembolic events survived. All patients with events had received thromboprophylaxis during the hospital stay.

### Standard biochemistry

The results of routine biochemical measurements are presented in Table 2. Median platelet count declined from admission to 72 h post admission along with a general INR and APTT prolongation, increasing fibrinogen and slightly elevated D-dimer. CRP increased from admission to 72 h whereas the leucocyte counts declined. Hemoglobin also fell significantly from admission to 72 h post admission.

**Table 2:** Laboratory parameters among trauma patients during the initial 72 h of hospital stay. Values displayed as median (interquartile range).

	Admission, n = 418	15 h, n = 280	72 h, n = 144
Platelet count, × 10^9^/L *Ref Female*: 165–400 × 10^9^/L *Ref Male*: 145 – 350 × 10^9^/L	231 (195–276)	198 (167–235)***	176 (138–210)***/###
Immature platelet fraction *Ref*: 0.016–0.126	0.034 (0.025–0.049)	0.034(0.025–0.048)ns	0.039 (0.029–0.058)*/#
INR *Ref*: < 1.2	1.1 (1–1.1)	1.2 (1.1–1.3)***	1.1 (1.1–1.2)***/###
APTT, sec *Ref*: 20–29 s	23 (22–25)	25 (23–27)***	26 (24–29)***/###
D-dimer, mg/L *Ref*: < 0.50 mg/L	2.6 (0.6–13.2)	3.4 (1.2–10.0)***	2.9 (1.8–4.5)***/###
Fibrinogen, µmol/L *Ref*: 5.5–12.0 µmol/L	8.0 (6.6–9.7)	8.2 (6.9–9.8)**	15.4 (13.0–18.6)***/###
Antithrombin, IU/L Ref: 0.80–1.20 × 10^3^ IU/L	1.05 (0.96–1.15)	0.96 (0.88–1.04)***	0.99 (0.90–1.09)ns/###
Hematocrite fraction *Ref Female*: 0.35–0.46 *Ref Male*: 0.40–0.50	0.41 (0.39–0.44)		
Hemoglobin, mmol/L *Ref Female*: 7.3–9.5 mmol/L *Ref Male*: 8.3–10.5 mmol/L	8.7 (8.0–9.3)	7.6 (6.8–8.4)***	6.6 (5.6–7.5)***/###
Leukocytes, × 10^9^/L *Ref*: 3.5–10.0 × 10^9^/L	10.2 (7.5–13.7)	9.9 (7.1–11.7)***	9.0 (7.1–11.2)***/###
CRP, mg/L *Ref*: < 3.0 mg/L	1.2 (0.6–3.1)	22.0 (8.5–44.8)***	100.6 (51.9–173.2)***/###
ALT, U/L *Ref: Female*: 10–45 U/L *Ref: Male*: 10–70 U/L	27 (19–45)		
Creatinine, µmol/L *Ref: Female*: 45–90 µmol/L *Ref: Male*: 60–105 µmol/L	78 (67–89)		
Alkaline phosphatase, U/L *Ref Female*: 45–115 U/L *Ref Male*: 60–235 U/L	68 (56–82)		

Abbreviations: *ALT* Alanine aminotransferase, *APTT* Activated partial thromboplastin time, *CRP* C-reactive protein, *INR* International Normalized Ratio, *Ref* Reference range. */**/***: *p* < 0.05/0.01/0.001 against admission level. #/##/###: *p* < 0.05/0.01/0.001 against 15 h levels.

### Thrombin generation

**Figure 2:**
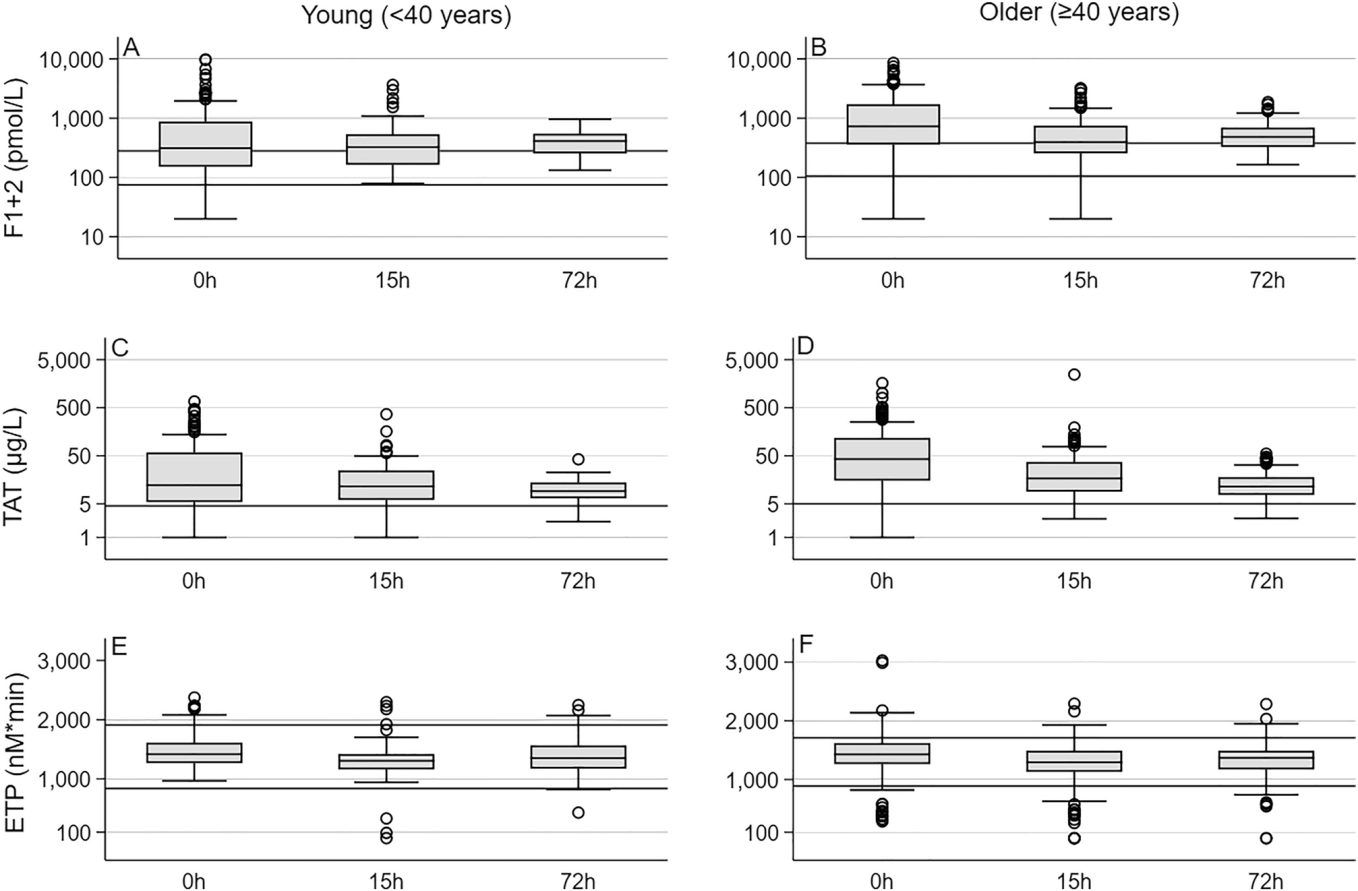
Prothrombin fragment F1+2 (F1+2), thrombin-antithrombin (TAT) complexes, and endogenous thrombin potential (ETP) on admission to hospital (0 h) and after 15 and 72 h hospital stay. (**A**) and (**B**): F1+2 levels were above the age-dependent reference intervals in 55% (95% CI 47–62%) of the young and 74% (95% CI 68–80%) of the older patients on admission. Compared with admission levels, relative F1+2 levels 15 and 72 h after the admission were 1.08 (95% CI 0.50–1.67,) and 0.49 (95% CI 0.32–0.66) among the young and 0.95 (95% CI 0.33–1.58) and 1.23 (95% CI − 0.22–2.68) in the older patients. 2.3% (95% CI 0.1–4.6) of the young and 1.2% (95% CI 0.2–2.7%) of the old presented with levels below the reference interval. (**C**) and (**D**): TAT complex levels on admission were above the age-dependent reference intervals in 79% (95% CI 73–85%) of the young and 91% (95% CI 88–95%) of the older patients. Compared with admission levels, relative TAT complex levels 15 and 72 h after the admission were 0.73 (95% CI 0.42–1.05) and 0.26 (95% CI 0.15–0.38) in the young and 1.11 (95% CI 0.30–1.93) and 0.36 (95% CI 0.14–0.58) in the older patients. (**E**) and (**F**): The ETP on admission was above the age-dependent reference interval in 7.0% (95% CI 3.2–10.8%) of the young and 15.5% (95% CI 10.9–20.1) of the older patients. None of the younger patients presented with an ETP below the reference interval while this occurred in 7.6% (95% CI 4.2–10.9) of the older patients. Relative to admission levels, the ETP at 15 and 72 h were 0.92% (95% CI 88.4–95.5) and 0.99% (95% CI 0.93–1.04) in the younger patients and 0.93% (95% CI 0.89–0.96) and 0.99 (95% CI 0.92–1.06) in the older patients. Boxes are median with interquartile range and whiskers are 2.5 and 97.5% percentils. Thick horizontal lines indicate age-dependent reference intervals for each parameter.

Plasma levels of F1+2 and TAT complexes were above the age-dependent reference intervals on admission to hospital as well as after 15 and 72 h hospital stay (Fig. 2a–d). The elevations were more pronounced among the older than the younger patients. In the younger patients, F1+2 and TAT complex levels fell from admission to 72 h, whereas only TAT levels decreased in the older patients. Significant reductions in F1+2 were not observed.

Admission levels of endogenous thrombin potential were increased in both age groups but more so among the older than the younger patients (Fig. 2e, f). Among the younger patients, none of the patients had an endogenous thrombin potential below the age-dependent reference interval while this occurred in 7.6% (95% CI 4.2–10.9) of the older patients. Average endogenous thrombin potential levels decreased from admission to 15 h in both age groups. The admission levels of peak thrombin were above the age-dependent reference interval in 36% (95% CI 30–42) of the older patients but were not elevated among the younger patients [2.9% (95% CI 0.4–5.4), *p* = 0.36). Among the younger patients none had a peak thrombin below the age-dependent reference interval at admission whereas this occurred in 4.2% (95% CI: 1.7 to 6.8, p = 0.05) of the older patients. Mean peak thrombin levels decreased from admission to 15 h in both the young [15 h/admission = 0.84 (95% CI: 0.80 to 0.88)] and older [15 h/admission = 0.88 (95% CI 0.83–0.92)] patients.

Admission levels of F1+2 and TAT were closely correlated (rho = 0.89, *p* < 0.0001), while endogenous thrombin potential was not correlated with F1+2 (rho = 0.00, *p* = 0.93) nor TAT (rho =  − 0.08, *p* = 0.12). Peak thrombin was weakly correlated with F1+2 (rho = 0.13, *p* = 0.01) and TAT (rho = 0.11, *p* = 0.03).

### Age-dependent thrombin generation

The association of admission levels of F1+2, TAT complexes and endogenous thrombin potential with trauma severity was explored in a multivariable linear regression model controlling for patient age (Table 3). The ISS score was positively and independently associated with plasma F1+2 and TAT complex levels but not with endogenous thrombin potential. For F1+2, an interaction between age and ISS score was uncovered. While this interaction was not found for TAT complex levels or endogenous thrombin potential, all parameters were further explored in an age-stratified analysis. It confirmed a stronger association between plasma F1+2 and ISS score in ter younger group of patients than in the group of older patients. To explore this further, the association of F1+2, TAT complexes and endogenous thrombin potential with dichotomized ISS score was analyzed. The plasma F1+2 and TAT complexes among patients admitted with an ISS score above 15 were increased by a factor 3.76 and 5.21, respectively, compared with those with ISS score of 15 or below, and this association was stronger among the younger than the older patients for both parameters (Table 3). Endogenous thrombin potential was not associated with trauma severity in any analysis.

**Table 3:** Prothrombin fragment F1+2, thrombin-antithrombin complex levels and endogenous thrombin potential in relation to injury severity.

	Per 10 points ISS score			Severe (ISS > 15) vs. light (ISS ≤ 15) injury		
	F1+2	TAT	ETP	F1+2	TAT	ETP
All patients^a^	1.78 (1.64–1.93)	2.19 (1.97–2.44)	3.63 (− 27.1–34.4)	3.76 (3.04–4.65)	5.21 (3.90–6.94)	42.5 (− 34.3–119.2)
Young patients (18–39 years)	1.98 (1.75 to 2.23)	2.31 (1.96–2.73)	− 8.3 (− 47.1–30.5)	6.64 (4.55–9.68)	10.52 (6.36–17.40)	− 33.6 (− 148.5–81.3)
Older patients (≥ 40 years)	1.64 (1.47–1.83)	2.12 (1.84–2.45)	− 5.01 (− 51.9–41.8)	2.92 (2.27–4.76)	3.86 (2.71–5.49)	46.0 (− 59.9–151.9)

F1+2, TAT complex levels and ETP in relation to ISS score. Results for all patients and by age-group displayed. Linear regression with ln(F1+2), ln(TAT) or ETP as dependent variable. Regression coefficients (beta coefficients) with 95% confidence interval presented. The ln-tranformed regression coefficients are back-transformed to linear scale before presentation.

Abbreviations: *ETP* endogenous thrombin potential, *F1+2* prothrombin fragment F1+2, *ISS* Injury Severity Score, *TAT* Thrombin-antithrombin complex levels.

^a^Adjusted for age.

### Thrombin generation and mortality

The charateristics of patients subcrumbing to their injuries are displayed in Table 4. In a logistic regression analysis adjusted for age and ISS score, each 1000 pmol/L increase in F1+2 and each 100 µg/L increase in TAT complex levels were independently associated with an odds ratio (OR) for death [(F1+2; OR = 1.39 (95% CI 1.06–1.82) and TAT; OR = 1.35 (95% CI 1.00–1.82)]. By contrast, changes in endogenous thrombin potential were not associated with 30 days mortality [OR = 0.99 (95% CI 0.99–1.0)].

**Table 4:** Admission charateristics of patients dying/surviving the initial 30 days after trauma.

	Dying, n = 15	Surviviors, n = 403	P-value
Age, years	59 ± 25	45 ± 20	0.01
Sex (male/female), n	12/3	268/135	0.28
ISS	25 ± 18	10 ± 10	< 0.001
F1+2, pmol/L	2815.1 ± 2668.7	1001.1 ± 1287.9	< 0.001
TAT, µg/L	274.3 ± 402.8	69.0 ± 111.6	< 0.001
ETP, nM*min	1302.3 ± 434.6	1438 ± 334.9	0.13
Thromboembolic events (n)	4	8	

Data presented as mean ± sd unless stated otherwise.

Abbreviations: *ETP* endogenous thrombin potential, *F1+2* prothrombin fragment F1+2, *ISS* Injury Severity Score, *TAT* Thrombin-antithrombin complex levels.

**Figure 3:**
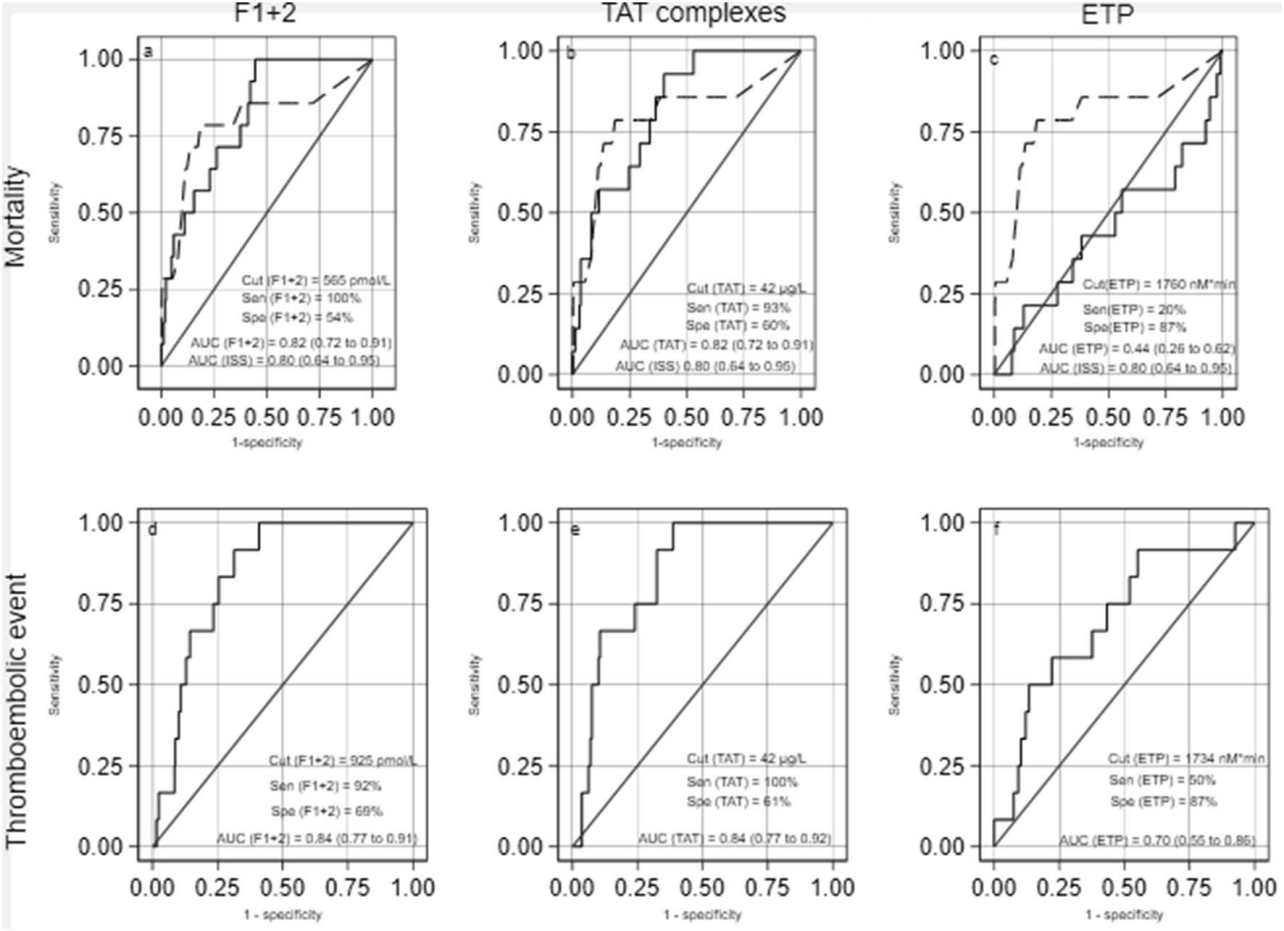
Reciever operating characteristic (ROC) curve analysis of the ability of admission levels of F1+2, TAT complexes and ETP to separate dying/surviving and thromboembolic events 30 days after multiple trauma.  A–C: Separation of dying/surviving patients. D–F: Separation of thromboembolic events. Area under the curve (AUC) as well as sensitivity (Sen) and specificity (Spe) at defined cut points (Cut) by youden index shown. Solid lines indicate F1+2, TAT complex levels and ETP. Punctured lines indicate ISS score. The AUC of F1+2, TAT complexes and ETP to separate dying from surviving patients was compared with ISS score. F1+2 versus ISS (*p* = 0.76), TAT complexes versus ISS (*p* = 0.80) and ETP versus ISS (*p* = 0.02). The Youden Index identified a sensitivity of 79% and specificity of 81% for ISS at a cut point of 17.5. Abbreviations: *ETP* endogenous thrombin potential, *F1+2* prothrombin fragment F1+2, *ISS* Injury Severity Score, *TAT* Thrombin-antithrombin complex levels.

In a ROC curve analysis, the ISS score separated dying/surviving patients with an area under the curve (AUC) of 0.80 (95% CI 0.64–0.96). An ISS score of 17.5 provided 79% sensitivity and 81% specificity for this separation. Admission levels of F1+2 and TAT complexes separated dying/surviving patients with an AUC comparable to the ISS score (*p* > 0.76 for both) whereas the endogenous thrombin potential showed no discriminative ability (Fig. 3a–c). At the cutpoint of 565 pmol/L, plasma F1+2 levels separated dying patients from survivning patients with a sensitivity and specificity of 100% and 54%. The corresponding cutpoint for TAT complex levels was 42 µg/L, which separated dying and survivning patients with 93% sensitivity and 60% specificity.

### Thrombin generation and thromboembolic events

Charateristics of patients experiencing thromboembolic events are presented in Table 5. After adjustment for age and ISS score, each 1000 pmol/L increase in F1+2 at admission was independently associated with development of any thromboembolic event, OR = 1.46 (95% CI 1.07–2.00). A 100 µg/L increase TAT complex levels at admission was associated with an OR of 1.42 (95% CI 1.03–1.96) and 100 nM*min increase in endogenous thrombin potential was associated with an OR = 1.32 (95% CI 1.22–1.55) for any thromboembolic event.

**Table 5:** Characteristics of patients with thromboembolic events.

	Any event, n = 12	Venous event, n = 7	Arterial event, n = 5	No event, n = 395
Age, years	64 ± 14 *P* = 0.001	61 ± 17, *P* = 0.03	68 ± 9 *P* = 0.01	45 ± 20
Sex (male/female), n	10/2 *P* = 0.21	6/1, *P* = 0.28	4/1 *P* = 0.51	261/134
ISS	17 ± 9 *P* = 0.03	16 ± 10, *P* = 0.16	18 ± 9 *P* = 0.08	10 ± 10
Dead, n	4	0	4	0
Thromboprofylaksis	12	7	5	131
F1+2, pmol/L	2302.1 ± 1438.8 *P* < 0.001	2516.1 ± 576.1 *P* <0.01	2002.8 ± 1419.9 *P* = 0.07	971.3 ± 1269.7
TAT, µg/L	170.7 ± 100.8 *P* < 0.01	173.6 ± 89.1 *P* = 0.01	166.5 ± 126.4 *P* = 0.05	66.8 ± 111.1
ETP, nM*min	1702.9 ± 477.1 = <0.01	1682.6 ± 160.8 *P* = 0.05	1731.5 ± 765.2 *P* = 0.05	1430.2 ± 326.2

Admission charateristics of patients experiencing symptomatic thromboembolic events within 30 days of the trauma. Any event are pooled data for venous and arterial events. Each group (any, venous, arterial) are compared with “no-event”. The “no-event” group excludes non-survivors.

Abbreviations: *ETP* endogenous thrombin potential, *F1+2* prothrombin fragment F1+2; ISS Injury Severity Score; TAT: Thrombin-antithrombin complex levels.

Data presented as mean ± sd unless stated otherwise.

In a ROC curve analysis, admission levels of F1+2 and TAT complexes separated patients experiencing thromboembolic events from those who did not suffer such events with a high AUC (Fig. 3d–f). In comparison, endogenous thrombin potential had a modest ability to separate these groups. The Youden Index provided high sensitivities of F1+2 and TAT complex levels to identify patients with symptomatic thromboembolic events (Fig. 3d, e).

## Discussion

In this study, we observed age-dependent differences in in vivo thrombin generation after trauma. Younger patients responded stronger to increasing injury severity and displayed more rapid reductions in in vivo thrombin generation, reflected by F1+2 and TAT complex levels, along time after trauma than older patients. In comparision, ex vivo thrombin generation was not associated with injury severity, but reduced levels on admission were observed among older patients only. Intrigingly, admission levels of biomarkers of in vivo thrombin generation separated patients dying/surviving 30 days after the trauma with an AUC comparable to ISS score and with high sensitivities suggesting a predictive ability. Furthermore, thrombin generation markers also predicted symptomatic thromboembolic events.

Our study extends established knowledged of the association between trauma severity and thrombin generation^9,16–18^ as we identified age dependency in the elicted thrombin response. While age-dependent reference intervals of haemostatic biomarkers are widely used^19,20^, knowledge of differences in response to injuries of comparable severity is scarce. In a previous study, Johansson et al. reported age-dependent differences in catecholamine, leucocyte, and platelet response to trauma, while F1+2 and TAT complexes were not differentially regulated^13^. However, the study was likely underpowered to detect differences in in vivo thrombin generation^13^. Further supporting our data, Kale et al. demonstrated that older patients who recovered clinically from sepsis had significantly higher D-dimer and TAT complex levels upon discharge from hospital than younger patients^14^. Comparable results have been observed after treatment for community-acquired pneumonia^15^. As such, our data clearly demonstrate that younger patients mount a stronger and more dynamic response to trauma than older patients. Furthermore, reductions in endogenous thrombin potential at admission were observed only among older patients in our cohort. Comparable results have been presented previously^21^ and may suggest that older patients, in turn, easilier get depleated and becomes prone to exanguination^10,11^.

We observed an independently predictive value of biomarkers of in vivo thrombin generation for 30-day mortality that was at least at the level of the ISS score. While the lethal effect of early coagulopathy in trauma is well-known^22^, it has been mostly associated with prolongation of haemostasis meaures such as the prothrombin time and partial thromboplastin^3^. However, as biomarkers of in vivo thrombin generation will accumulate in the circulation, it is comprehensible that an association with trauma outcome exsists but as far as to our knowledged this has not been previously evaluated. In a post-hoc analysis, Levi et al. reported a dose–response relationship between F1+2 and TAT complexes with mortality in sepsis-associated coagulopathy^23^ which substantiates our findings. The AUC, sensitivity and specificity of ISS to predict death in our cohort are at levels comparable to that reported by others^24^. This is compelling because F1+2 and TAT complex levels can easily be measured directly upon patient admission, whereas ISS is retrospectively calculated. Therefore, measurements of F1+2 and TAT complexes could be valuable in the initial patients evaluation and risk stratification. Further prospective clinical studies are needed to explore this strategy.

The clinical course of post-trauma care is often complicated by vascular events, and the presence of early coagulopathy is a strong predictor for subsequent thromboembolic events^3,8,25^. International guidelines are increasingly focused on the need for chemical thromboprophylaxis, except to those with lighter injuries which may be arbitary defined by an ISS ≤ 10 or < 24 h of hospital stay^26^. Consequently, rates of coverage with chemical thromboprophylaxis are often higher than in our study^8^. However, this difference is likely explained by the fact that many trauma studies are focused on severely injured patients while our cohort contain many patients with light injuries and a shorter hospital stay. Our data are in line with the established association between increased ex vivo thrombin generation at admission and a risk of subsequent thromboembolic events^25^. The association to biomarkers of in vivo thrombin generation is less established but F1+2 concentrations predict development of venous thromboembolism in surgical patients^27^, patients with cancer^28^, and among patients with severe infections^29^. We observed 12 symptomatic thromboembolic events that occurred among patients treated with low molecular weight heparin thromboprophylaxis. These patients were older and more severely injured than those without events and were as such were more prone to develop thromboembolic complications^6,8^. These patients had elevated in vivo thrombin generation throughout the study whereas their ex vivo endogenous thrombin potential was elevated only on admission to hospital. Since, the observed thromboembolic events were developed despite thromboprophylaxis, it is tempting to speculate if excessively elevated biomarkers of in vivo or ex vivo thrombin generation should lead to intensified antithrombotic treatment. With the current emphasis on early thromboprophylaxis in trauma patients^26^, it can be speculated that the timing of profylaxis may have influenced our results. In our study, the exact timing of the prophylaxis was not registred and it cannot be ruled out that the development of thromboembolic complications has been affected (positively or negatively) by the used strategy. However, we analyzed only the association between thrombin generation measured before the initiation of thromboprophylaxis and subsequent thromboembolic events. As such, the observed association would be applicable to any trauma patient while the magnitude of the association may be modified by different prophylaxis strategies. Therefore, further studies are needed to confirm the observed association and to explore how the biomarkers can be used in timing and dosing of pharmacologic thromboprophylaxis.

The present study has some limitations to consider. While being among the larger end prospective clinical trauma studies with very little drop out, it has few thrombotic events and deaths. This is a weakness from a scientific point of view, but it reflects the actual clinical situation in our hospital. We excluded patients dead on arrival or transferred directly for neurosurgery. This may have lead to underestimation of the significance of thrombin generation as well as a reduction of the prevalence of neurosurgical injuries in our cohort. However, only few patients were missed on these grounds, and it is judged to have had limited impact on the results. The cohort consists mainly of blunt traumas which warrents caution when compared to previous studies. However, it reflects the trauma population in Denmark and provides an unselected picture of our trauma patients. Due to local registration procedures, we were unable to retrive some key parameters, foremost acid–base balance or base excess and resuscitation volume. However, the prevalence of shock on admission was low and the admission hematocrite level within the reference interval which suggests that large deviations in acid–base balance or massive volume resuscitation have not occurred. In our study, thromboembolic events were registred only in symptomatic patients, and we may therefore have missed subclinical events. This approach was used since we studied a mixed trauma population and discharged patients could not be systematically followed. However, according to national guidelines, patients with symptoms of thromboembolic disease are referred to hospital for investigation, and the majority of events developed within 30 days after discharge would have been diagnosed.

In conclusion, younger patients mounted a stronger and more dynamic in vivo thrombin response to major blunt trauma than older patients who, by contrast, were more prone to depletion of ex vivo thrombin generation. Admission levels of F1+2 concentration and TAT complex had a strong ability to predict death and/or thromboembolic events 30 days after the injury. This may suggest that biomarkers of in vivo thrombin generation could prove valuable in clinical risk stratification of trauma patients.

## Materials and methods

### Study population

Patients aged 18 years or above, admitted to the level I trauma center of Aarhus University Hospital, Denmark, in the period from March 2017 to March 2018 were evaluated for inclusion. Patients were excluded from the study if: dead on arrival to the trauma center; pregnant; not fulfilling the Advanced Trauma Life Support (ATLS) criteria for trauma team activation^30^; blood sampling proved practically impossible; the patient or the substitute declined or withdrew consent. Patients readmitted during the study period were included in the study only once for the admission associated with most collected samples.

The study was conducted in accordance with the Helsinki declaration and was approved by the local ethical committee (1-10-72-205-16 and 1-10-72-204-16) and the Danish Data Protection Authority (1-16-02-452-16). All patients or their next of kin provided written informed consent to participate in the study. In the present study, informed consent was obtained as soon as possible after collection of the admission blood sample. For patients incapable of providing consent, this was obtained from a physician not involved in the study or treatment of the patient as well as from the patients next-of-kin. Patients who gained their ability to consent within 72 h after admission were asked to confirm or deny the informed consent.

### Data collection – the trauma registry

The local trauma registry contains information on all patients admitted to the level I trauma center of Aarhus University Hospital. It was used to gather information on the total number of admissions in the study period, time of injury, type of injury, and prehospital treatment interventions. The trauma registry was also used to obtain information on vital signs (heart rate, respiratory frequency, blood pressure and temperature) upon arrival to the trauma resuscitation room. The Abbreviated Injury Severity (AIS) score was obtained from the registry and the Injury Severity Score (ISS)^31^ subsequently calculated. Patients with a ISS > 15 were classified as severely injuried, and those with a Shock Index ≥ 0.8 were considered in circulatory shock^32^. The information contained in the trauma registry was systematically collected by a dedicated team of junior doctors. The information on prehospital parameters contained in the trauma registry was extracted from the prehospital medical records which are kept by the prehospital doctors with special training in prehospital patient treatment. The registry information on patient status on arrival to the trauma resuscitation room was extracted from the trauma chart.

### Data collection—medical records

Danish hospitals keep an electronic medical record covering all clinical and paraclinical patient information. These medical records were used to collect information on the time of hospital admission and the time of blood sampling, as well as age, sex and pre-injury antiplatelet, anticoagulant or antihypertensive treatment. Patients were registered as not receiving this type of treatment if the information was absent. Blood transfusions were registered if described in the medical record or in the blood bank laboratory system within the first 72 h of admission. A transfusion was categorized as massive transfusion if more than 10 units of red blood cells and/or fresh frozen plasma were administered within the first 72 h. Information on administration of low molecular weight heparin was classified as early if initiated within 72 h after admission and as late administration if after 72 h. The information on in-hospital administration of tranexamic acid was collected from the medical records and combined with information on prehospital administration obtained from the trauma registry.

Information on surgical procedures performed within the first 72 h of the hospital stay was collected from the medical records. If more surgical procedures within the same surgical specialty and anatomical area were performed on the same day, they were registered as one procedure only. E.g., osteosynthesis on right humerus and antebrachium within the same day was registered as one orthopedic procedure, whereas a simultaneous humerus and femur surgery was registered as two orthopedic procedures. Repeated surgical procedures in the same anatomical area were registered as individual surgeries if performed on different days. One round of surgery was defined as one or multiple procedures performed within the same day. Repeated rounds of surgery were defined as two or more consecutive surgeries within the first 72 h of admission.

The length of hospital stay was defined as the duration in days and hours from admission to discharge according to the medical record. The patients were followed up by chart review 30 days after the admission. Information on mortality and symptomatic thromboembolic events was registered. Information on symptomatic thromboembolic events was also obtained from the medical records. Venous thromboembolic events were diagnosed by computed tomography or ultrasound and arterial events by angiography.

### Blood samples

Blood samples were collected in the trauma resuscitation room upon arrival, 15 ± 3 h after admission, and 72 ± 6 h after admission. Patients discharged or transferred to another hospital within 72 h after the admission left the study, and further blood sampling was not attempted. The blood was collected from the arterial line or by venous puncture by a dedicated team of certified technicians. Blood samples were drawn into either citrated-, EDTA-, heparin- or serum tubes (BD Vacutainer^®^, Becton, Dickinson and Company, Franklin Lakes, NJ, USA). The samples were transported to the laboratory at room temperature. Samples for chemistry, coagulation and hematologic analysis were processed within one hour and according to the guidelines for our clinical laboratory. Samples for subsequent thrombin generation assay (TGA) analysis were centrifuged within one hour of collection at 3000× g, 25 min. at room temperature to obtain platelet poor plasma (PPP). Subsequently, the plasma was aliquoted and frozen at − 80 °C until further analysis.

### Biochemical analysis

Chemistry [plasma-glucose, creatinine, C-reactive protein (CRP), alkaline phosphatase (ALP) and alanine aminotransferase (ALT)], coagulation parameters [antithrombin, fibrin D-dimer, fibrinogen, activated partial thromboplastin time (APTT) and International Normalized Ratio (INR)], and hematologic parameters [hemoglobin, hematocrite, leukocyte count, platelet count and immature platelet fraction] were measured at the Department of Clinical Biochemistry at Aarhus University Hospital using assays validated for routine clinical use. Chemistry parameters were measured using Roche Cobas 6000, coagulation parameters on Siemens CS2100, and hematology parameters was analyzed by Sysmex XE-5000.

In vivo thrombin generation was measured by determination of prothrombin fragment F1+2 (F1+2) and thrombin-antithrombin (TAT) complex levels. F1+2 concentrations were analyzed in 3.2% citrated plasma by the Enzygnost^®^ F1+2 (monoclonal) ELISA kit (SIEMENS Healthcare Diagnostics Products GmbH, Marburg, Germany) according to the manufacturer's instructions and as published previously^20^. Briefly, the samples were thawed at room temperature and batch analyzed in duplicate. According to the manufacturer, the measuring range of the assay is 20–1200 pmol/L. The intra-assay coefficient of variation (CV) is reported to be 3.6–5.5% in the range from 40–1170 pmol/L and the inter-assay CV 4.4–11.2%. The precision is stated to be 6.8–11.8%. Samples above the upper measurement range were diluted a maximum of 1:20 according to the manufacturer’s instructions. Samples below the lower measurement range were rerun and, if remaining low, they were assigned the lower measurement value. Maximum CV allowed between duplicates was 10%, and the results are reported as the mean of the duplicate values.

TAT complex levels were analyzed in 3.2% citrated plasma by the Enzygnost^®^ TAT micro ELISA assay (SIEMENS Healthcare Diagnostics) according to the manufactures instructions and as published previously^20^. According to the manufacturer, intra and inter assay CVs are 6% and 9% and the measurement range is 2.0–60 µg/L. Samples were batch analyzed in duplicate and are reported as the mean of duplicates. A maximum CV of 10% between duplicates was allowed. The reference intervals for F1+2 and TAT were determined on the basis of previously published data^20^. The F1+2 reference interval was determined as the 95% prediction interval for the defined age groups, whereas the reference interval for TAT complex levels was determined as the upper 97.5% centile after removal of outliers by Tukey`s fences using 3 × the interquartile range (iqr)^33^. The F1+2 reference intervals were 75–280 pmol/L and 105–378 pmol/L and the TAT reference intervals were 4.5 µg/L and 5.0 µg/L for the young and the old age groups, respectively.

Ex vivo thrombin generation was analysed with the calibrated automated thrombogram (Thrombinoscope BV, Masstricht, Netherlands). Citrated, platelet poor, plasma was thawed at 37 °C and centrifugated at 17,000 rcf, 3 min. at room temperature. Thrombin generation was measured in duplicate after addition of 5 pM tissue factor, 4 µM phospholipids, calcium chloride and FluCa (Thrombinoscope BV). The thrombogram was captured using a Flouroskan AscentTM Microplate Flourometer (Thermo ScientificTM, Waltham, MA, USA) using the Thrombinoscope software (Thrombinoscope BV). For this study, the endogenous thrombin potential (nM*min) and peak thrombin generation was recorded. The age-dependent reference intervals for the endogenous thrombin potential and peak thrombin generation for the young and older patients are 838–1908 nM*min and 888–1709 nM*min and 91–379 nM and 84–285 nM, respectively^34^.

### Statistical analysis

Data distribution was assessed by inspection of inverse QQ-plots. Data are presented as absolute numbers and percentages, means with standard deviation (sd) or 95% confidence intervals (CI), or as medians with iqr, depending on the data distribution. Categorical data were analyzed by the chi square test. Comparison of continuous data was done by one- or two-sample Student’s t-test, the Mann–Whitney U-test as appropriate. Comparison of repeated biochemical measures was performed by Kruskal–Wallis test followed by Wilcoxon Signed Rank test. Correlations were analyzed by Spearman’s rho. The percentage of patients in each age group above or below the reference limits was calculated and compared to the fraction expected to exceed the reference limit (2.5%) by a one-sample t-test. To assess changes in F1+2, TAT complex levels and endogenous thrombin potential after admission, levels at 15 and 72 h were expressed relatively to admission levels and analyzed by a one-sample t-test. The influence of injury severity and trauma type on admission levels of F1+2, TAT complexes and endogenous thrombin potential was analyzed in a multivariable regression model controlling for age. The F1+2 and TAT complex levels were positively skewed and thus ln-transformed before the statistical analysis. Results were transformed back to the linear scale before presentation. In the initial analyses, ln(F1+2), ln(TAT) and endogenous thrombin potential were the dependent variables, whereas ISS, trauma type, and age were the independent variables. Interactions between age and ISS as well as ISS and trauma type were included in the analyses. To further explore the effect of injury severity, ISS was dichotomized 0–15 and 16–75 and analyzed through a similar approach. The predictive value of plasma F1+2, TAT complex and endogenous thrombin potential on 30 day mortality and thromboembolic events was explored in a logistic regression model controlling for age and ISS score. The analysis of the discriminative ability of plasma F1+2, TAT complex levels and endogenous thrombin potential to separate dying and surviving patients as well as patients with or without thromboembolic events was explored by receiver operating characteristics (ROC) curve analysis, and the sensitivity and specificity at the mathematically optimal cut-point was calculated by the Youden index. The equality of ROC areas for ISS against F1+2, TAT complexes and endogenous thrombin potential was compared by the method described by DeLong et al.^35^. The admission level of F1+2, TAT complexes and endogenous thrombin potential was missing in two, three and two of the dying patients and in one, two and one of the patients with thromboembolic events, respectively. For the analysis of mortality and thrombotic events, these missing values were replaced by the 15 h values. For all statistical analyses, the alpha level was set at 0.05. The statistical work was carried out using the STATA 16.1 statistical package.

## Data Availability

Data will be made available upon reasonable request. Requests should be addressed to the corresponding author: Claus VB Hviid.
